# Therapeutic Strategies in Huntington’s Disease: From Genetic Defect to Gene Therapy

**DOI:** 10.3390/biomedicines10081895

**Published:** 2022-08-05

**Authors:** Anamaria Jurcau, Maria Carolina Jurcau

**Affiliations:** 1 Department of Psycho-Neurosciences and Rehabilitation, Faculty of Medicine and Pharmacy, University of Oradea, 410073 Oradea, Romania; 2 Neurology 3 Ward, Clinical Emergency County Hospital Oradea, 410169 Oradea, Romania; 3 Faculty of Medicine and Pharmacy, University of Oradea, 410073 Oradea, Romania

**Keywords:** Huntington’s disease, mutant huntingtin, antisense oligonucleotides, RNA interference, CRISPR/Cas9, zinc finger proteins, stem cell therapies

## Abstract

Despite the identification of an expanded CAG repeat on exon 1 of the huntingtin gene located on chromosome 1 as the genetic defect causing Huntington’s disease almost 30 years ago, currently approved therapies provide only limited symptomatic relief and do not influence the age of onset or disease progression rate. Research has identified various intricate pathogenic cascades which lead to neuronal degeneration, but therapies interfering with these mechanisms have been marked by many failures and remain to be validated. Exciting new opportunities are opened by the emerging techniques which target the mutant protein DNA and RNA, allowing for “gene editing”. Although some issues relating to “off-target” effects or immune-mediated side effects need to be solved, these strategies, combined with stem cell therapies and more traditional approaches targeting specific pathogenic cascades, such as excitotoxicity and bioavailability of neurotrophic factors, could lead to significant improvement of the outcomes of treated Huntington’s disease patients.

## 1. Introduction

A detailed description of the clinical manifestations of Huntington’s disease (HD) was given by George Huntington in a paper published in 1872, who also noticed the mode of transmission, later known as autosomal dominant [1]. Following the description of the disease, several cases were identified, with a clustering of HD cases identified by Dr. Amerigo Negrette around Lake Maracaibo, in Venezuela [1], where a group of researchers identified the defective gene to be located in the first exon of the huntingtin gene on chromosome 4, which contained an expanded unstable CAG (cytosine-adenine-guanine) repeat [2]. The normal allele has less than 27 CAG repeats, while individuals with 40 or more repeats will develop the disease with full penetrance. Individuals carrying between 27 and 35 CAG repeats usually do not develop the disease but may transmit it to their offspring.

The gene product, huntingtin (Htt), is a protein composed of 3144 amino acids with a polyglutamine (polyQ) domain at its NH_2_ terminus starting at amino acid position 18 and containing 11–34 glutamine residues [1], expressed at high levels in neurons, but also in astrocytes, oligodendrocytes, microglia [3], as well as in non-neural tissues [4].

In terms of pathology, HD is characterized by a striking atrophy of the caudate nucleus and putamen, with astrogliosis and cell loss in the striatum affecting especially the medium spiny neurons (MSNs), as well as neuronal loss in the cortical layers III, IV and VI in late stages [5].

The onset of the disease usually occurs in middle age, peaking between 35 and 50 years of age, when patients develop personality changes, involuntary choreic movements, variable degrees of rigidity, incoordination leading to progressive motor dysfunction, and a series of psychiatric symptoms, such as depression, anxiety, psychosis, obsessive-compulsive disorder, cognitive impairment progressing to dementia, and weight loss, eventually developing life-threatening complications from frequent falls, poor nutrition, infections, or swallowing difficulties leading to aspiration pneumonia [1]. Due to the higher risk of expansion of the unstable sequence of CAG repeats during mitosis in spermatogenesis compared to oogenesis, paternal transmission may result in earlier onset of symptoms (before the age of 20) and a more severe disease variant, associating parkinsonian features, dystonia, and seizures with a more rapid progression (juvenile HD) [1,5]. However, although a larger CAG repeat size correlates with earlier onset, the size of the CAG repeats explains only about 50% of the variance in age of onset. Several other factors contribute, such as a number of polymorphisms in genes encoding for glutamic acid, glutamate receptors, or other proteins and enzymes involved in the pathogenesis of HD, or the CAG repeat size in the normal allele [1].

## 2. Pathophysiology of Huntington’s Disease

Following the identification of the genetic defect, many groups of researchers, using chemical models [6], various genetic animal models [7], or complex cell lines mimicking the intercellular signaling and circuitry of the human brain [8], focused on unraveling the complex mechanisms through which the expression of an abnormal protein (mutant huntingtin, mHtt) leads to the massive cellular degeneration and the loss of synaptic activity in HD. Several vicious pathways have been identified [5], but the detailed description of these pathways is beyond the scope of this review, which is why they will be only briefly summarized below.

### 2.1. Excitotoxicity

The striatum receives massive glutamatergic input mainly from the cortex and the thalamus [9], with glutamate acting on N-methyl-D-aspartate receptors (NMDARs), α-amino-3-hydroxy-5-methyl-4-isoxazolepropionate receptors and kainate receptors. NMDARs are tetrameric complexes formed by two GluN1 (formerly known as NR1) and two GluN2 (or NR2) and/or GluN3 (or NR3) subunits [8]. Mutant Htt alters the transcription of the GLuN2B gene, leading to an altered structure and increased glutamate sensitivity of this receptor [10]. In addition, impaired interaction of mHtt with the scaffolding protein postsynaptic density 95 (PSD95) disturbs the distribution of the NMDARs, with an increased number of extrasynaptic receptors [10,11]. The binding of glutamate to extrasynaptic NMDARs is followed by cellular calcium overload, calcium which is buffered by mitochondria at the expense of increased oxidative stress, and dephosphorylation and inactivation of CREB (cAMP-responsive element-binding protein) with the promotion of pro-death gene expression, as opposed to stimulation of synaptic NMDARs, which leads to the expression of anti-apoptotic and pro-survival proteins via phosphorylation of CREB [12,13,14]. In addition, increased cytosolic calcium activates calpain, which cleaves the GLuN2B subunit of NMDARs, modulating its surface distribution and increasing the expression of extrasynaptic NMDARs [15] in a vicious cascade. Another effect of the increased cytosolic calcium concentration is calcineurin activation, which induces the dephosphorylation of huntingtin at Ser421, promotes the toxic role of mutant huntingtin, and activates caspase 3 through the intrinsic pathway, leading to neuronal apoptosis [16].

### 2.2. Impairments of the Proteostasis

Proteins are continuously synthesized as linear amino acid chains, which fold into three-dimensional structures assisted by chaperones. Misfolded proteins are either refolded into their correct structure or targeted for degradation via the ubiquitin-proteasome system (UPS) [17,18]. Mutant Htt aggregates sequester chaperones, decreasing their availability and enhancing abnormal protein folding [19]. A unique α-amine E2 enzyme, UBE3A, whose activity decreases with age, specifically targets Htt fragments for ubiquitylation and degradation and could explain the late age of onset of this inherited disease [8]. In addition, the eukaryotic proteasome cannot digest large stretches with 9–19 polyQ residues [20], leading to the overwhelming of the UPS system by aggregation-prone misfolded proteins [21].

Another way of disposing of accumulated misfolded proteins is autophagy, a process during which a portion of the cytosol containing damaged proteins or organelles are degraded by lysosomes [22], and whose importance increases with age, as the proteasomal system’s efficiency starts to fail [23]. An important role in the regulation of autophagy belongs to the striatal-selective G-protein Rhes, which binds to mHtt and stimulates its sumoylation, thereby augmenting mHtt toxicity [24]. Normally, Rhes prevents beclin-1 from its inhibitory binding to Bcl-2, thereby increasing mTOR (mammalian target of rapamycin)-independent autophagy. Mutant Htt aggregates sequester Rhes, thereby interfering with its action. Although mHtt aggregates also sequester mTOR, which normally inhibits autophagy, this compensatory mechanism is not sufficient to enable the neurons to cope with mHtt toxicity [24]. In addition, particular conformations of mHtt are resistant to autophagy [25].

### 2.3. Mitochondrial Dysfunction

To meet their high energy demands, neurons rely mainly on mitochondrial oxidative phosphorylation, which implies that the shape, size, and number of mitochondria in the cells must be regulated through mitochondrial fission and fusion, two opposite processes regulated by specific proteins: dynamin-related/-like protein 1 (Drp1) and dynamin2 (Dnm2) [26] for fission, and mitofusins 1 and 2 (Mfn1 and Mfn2) as well as optic atrophy 1 (OPA1) for mitochondrial fusion [27]. Mutant Htt, through interaction with Mfn2 and a calcineurin-related increase in mitochondrial translocation of Drp1, significantly alters mitochondrial morphology and dynamics [28].

Furthermore, direct interaction of mHtt aggregates with mitochondrial proteins leads to a decrease in the activity of the electron chain complexes, especially of complexes II, III, and IV, [5,29], and a reduction in the mitochondrial membrane potential, with the opening of the mitochondrial permeability transition pore and release of cytochrome c and apoptosis-inducing factor (AIF), which activate caspase-dependent and caspase-independent pathways of apoptosis [12].

Given the particular morphology of the neuron, mitochondria must be trafficked along neuronal outgrowths to meet the local energy demands, a process regulated by dynactin together with kinesin for anterograde transport and dynein for retrograde transport [18], anchored to the outer surface of mitochondria by Miro [30]. HAP1 (huntingtin-associated protein 1) interacts with both kinesin and dynein in regulating cargo transport on microtubules [31] and recruits glyceraldehyde-3-phosphate dehydrogenase (GAPDH) to provide the necessary energy for transport by producing ATP. The sequestration of GAPDH and HAP1 into mHtt aggregates significantly impairs mitochondrial trafficking [5].

Mitochondria also have a crucial role in maintaining the calcium homeostasis of the cell through interacting with the endoplasmic reticulum, which expresses inositol 1,4,5-triphosphate receptors gated by inositol 1,4,5-triphosphate (IP3) and cytosolic calcium. Mutant Htt binds to the carboxy-terminus of the IP3 receptor and increases IP3 receptor responsiveness, allowing Ca^2+^ release at lower IP3 concentrations [32]. Calcium released from the endoplasmic reticulum elevates the store-operated Ca^2+^ channel (SOC) response [33] and leads to dendritic spine loss [32], in addition to activating a series of enzymes, such as calpain and calcineurin (discussed above), which can ignite other vicious pathogenic cascades.

### 2.4. Oxidative Stress

Oxidative stress, defined as an imbalance between the production of reactive oxidative species (ROS) and the ability of a biological system to clear these molecules [34], has been increasingly implicated in the pathogenesis of a range of diseases, neurodegenerative ones included [14,22].

The main source of ROS are mitochondria, which even under normal circumstances allow about 2% of electrons to “leak” from the electron transport chain, serving to produce ROS. In the case of dysfunctional mitochondria, as shown above, the amount of ROS produced increases dramatically and further impairs mitochondrial function, leading to calcium overload and opening of the mitochondrial permeability transition pore, which triggers apoptosis [35].

ROS also damage proteins, lipids [36], and DNA, repaired mainly through base excision repair (BER), in which OGG1 (8-oxoguanine DNA glycosylase-1) is responsible for the excision of 8-oxoguanine. In a transgenic mouse model of HD, an age-dependent somatic CAG expansion has been demonstrated in the process of removing oxidized DNA bases, including in post-mitotic neurons, involving the activity of OGG1 [37]. Although this process is less important in the presence of less than 27 CAG repeats in exon 1 of the Htt gene, it may contribute to escalating the toxicity of mHtt with age if an increased number of CAG repeats are present from birth [37].

Furthermore, ROS can stimulate receptors on immune cells and elicit the secretion of various cytokines which regulate macrophage polarization towards the pro-inflammatory M1 phenotype, igniting neuroinflammation [38], the role of which in HD [39], amyotrophic lateral sclerosis [40], or even stroke [41] is increasingly emphasized.

### 2.5. Transcriptional Dysregulation

Research has shown that mHtt interacts with several transcription factors and coactivator factors, leading to impaired transcription or altered function of important proteins. Mutant Htt interacts with the CBP (CREB binding protein)/p300 dimer and inhibits its acetylase activity, which is essential for Nrf2 (nuclear factor-erythroid 2-related factor-2) cellular localization and stability [42], thereby interfering with the Nrf2-ARE (antioxidant response element) pathway which regulates the transcription of a variety of antioxidant proteins and enzymes [36]. Mutant Htt also inhibits the phosphorylation of CREB [43] and downregulates the transcription of peroxisome proliferator-activated receptor gamma coactivator-1α (PGC-1α) through interference with the CREB/TAF4 transcriptional pathway in striatal neurons [44]. For the repressor element 1(RE1)—silencing transcription factor (REST), mHtt promotes its nuclear translocation and enables its repressor effect on target genes, such as the brain-derived neurotrophic factor (BDNF) gene [45]. Furthermore, by binding to p53 and increasing its transcriptional activity, mHtt upregulates pro-apoptotic factors, such as Bcl2-associated X protein (BAX) and p53-upregulated modulator of apoptosis (PUMA) [46].

### 2.6. Impaired BDNF Synthesis and Transport

Striatal neurons express low amounts of BDNF mRNA, but striatal medium spiny neurons (MSNs) receive large amounts of BDNF, necessary for trophic support, from cortical neurons along the cortico-striatal tract [47]. The interaction of mHtt with REST leads to accumulation of the REST/NRSF (neuron-restrictive silencer factor) in the nucleus of cortical neurons and impairment of BDNF transcription [48].

Furthermore, the transport of vesicles containing BDNF to striatal MSNs is impaired due to the sequestration of the energy-supplying glyceraldehyde-3-phosphate dehydrogenase (GAPDH) and other adaptor proteins, such as huntingtin-associated protein 1 (HAP1) or dynein, into the mHtt aggregates [49,50]. In addition, the activation of axonal c-Jun amino-terminal kinase3 via stress-signaling kinase results in kinesin-1 phosphorylation and detachment of kinesin-1 and cargo from microtubules [49].

### 2.7. Dysfunctions of Glial Cells

Intracellular mHtt aggregates were identified in astrocytes as well [3], where they might downregulate the expression of Kir4.1 (inwardly rectifying K^+^) channels, leading to decreased membrane potential and conductance [51]. These changes alter the sensitivity of astrocytes to neuromediators and pH [52]. In addition, the observed reduction of EAAT2 both at the mRNA and protein level impairs the ability of astrocytes to clear excess glutamate [53], increasing excitotoxicity, although the involvement of mHtt in producing these impairments has not been clearly demonstrated.

**Figure 1:**
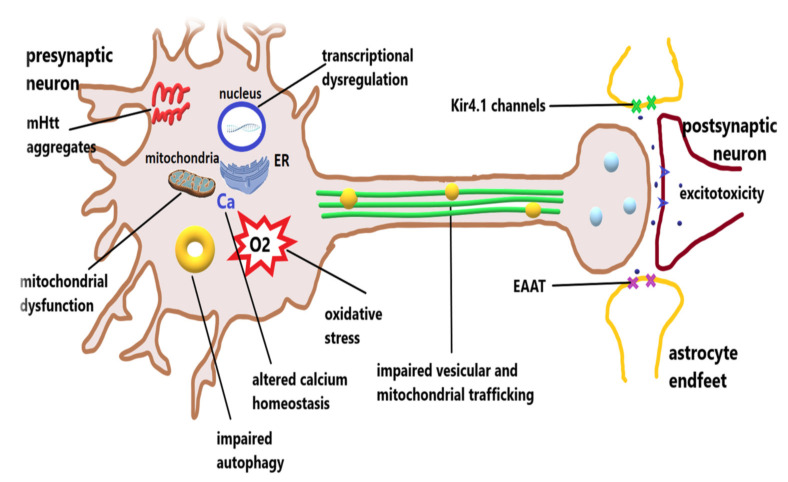
Pathophysiology of Huntington’s disease. The expression of abnormal, mutant huntingtin (mHtt) makes the protein prone to misfolding and aggregation, leading to impaired proteostasis. Autophagy is also defective in HD, caused by impaired recognition of cargo and vesicular trafficking. Mutant Htt interacts with a series of transcription factors, causing impaired transcription of a series of essential proteins, such as brain-derived neurotrophic factor, or proteins acting as pro- or anti-apoptotic factors. The mitochondrial dysfunction caused by mHtt, together with the altered calcium homeostasis, leads to increased oxidative stress, which further impairs mitochondrial function. Interaction of mHtt with motor proteins causes altered vesicular (yellow circles) and mitochondrial trafficking along the microtubules to distant sites of the neuron with deficient neuromediator release, especially of inhibitory neuromediators (light blue circles). In addition, deficient astrocytic function caused by the decreased function of inwardly rectifying K^+^ channels (Kir4.1) and diminished clearance of excess glutamate through reduction of excitatory amino acid transporter 2 (EAAT), together with altered function and distribution of N-methyl-D-aspartate receptors (NMDARs, blue arrow heads), creates the premises for excitotoxicity.

In oligodendrocytes, the binding of mHtt to the N-terminal myelin regulatory factor (nMYRF) and mHtt-induced altered PGC-1α expression leads to myelination deficits [54]. Figure 1 summarizes the multiple impairments contributing to the pathogenesis of Huntington’s disease.

### 2.8. Neuroinflammation in HD

By promoting the expression of genes encoding for pro-inflammatory cytokines, mHtt exaggerates the response of microglia to activating stimuli which are recognized via nucleotide-binding and oligomerization domain NOD-like receptors (NLRs) and toll-like receptors (TLRs) [41,55], triggering downstream pro-inflammatory cytokine expression. In addition, degenerating neurons, by releasing danger-associated molecular patterns (DAMPs), may further increase the inflammatory response [56]. In a vicious cascade, activated microglia produce cytokines and ROS, which activate astrocytic intracellular signaling pathways, such as the NF-κB, mitogen-activated protein kinase (MAPK), or the Janus kinase/signal transducer and activator of transcription (JAK/STAT), and lead to reactive astrocytes which further release cytokines and chemokines [57]. Table 1 summarizes the pathogenic cascades described above.

Many vicious cascades triggered by the expression of mHtt and contributing to the pathogenesis of Huntington’s disease have been described, including the following: NMDARs—N-methyl-D-aspartate receptors; PSD95—postsynaptic density 95; UPS—ubiquitin-proteasome system; Mfn2—mitofusin 2; Drp1—dynamin-related protein 1; MPTP—mitochondrial permeability transition pore; GAPDH—glyceraldehyde-3-phosphate dehydrogenase; HAP1—huntingtin-associated protein 1; IP3R—inositol 1,4,5-triphosphate receptor; ER—endoplasmic reticulum; ROS—reactive oxygen species; BER—base excision repair; OGG1—8-oxoguanine DNA glycosylase-1; CREB—cAMP-responsive element-binding protein; PGC-1α—peroxisome proliferator-activated receptor gamma coactivator-1α; REST—repressor element 1(RE1)—silencing transcription factor; NRSF—neuron-restrictive silencer factor; BDNF—brain-derived neurotrophic factor; Kir4.1 channels—inwardly rectifying K^+^ channels; nMYRF—N-terminal myelin regulatory factor; JAK/STAT pathway—Janus kinase/signal transducer and activator of transcription pathway; and MAPK—mitogen-activated protein kinase.

## 3. Therapeutic Strategies in Huntington’s Disease

To date, drugs currently used in the treatment of HD address the symptoms of the disease and aim to control the motor and behavioral abnormalities, but only have limited benefits and do not address disease progression [58]. The search for more efficient therapies has been marked by promising results in preclinical research and many clinical failures. The novel gene-silencing approaches would be the most logical strategies to be applied to HD carriers, thereby preventing the expression of the mutant protein and preventing all the downstream pathogenic cascades, but they are still in their infancy. Until the precise beneficial and unwanted effects are clarified, together with the proper delivery methods, it is likely that research targeting the described pathogenic cascades will continue. However, it appears that drugs directed against single mechanisms did not meet expectations, and a cocktail of drugs targeting multiple mechanisms would be more rewarding [36].

### 3.1. Therapeutic Strategies Targeting Pathogenic Cascades

Below we briefly summarize in Table 2 the preclinical and clinical results obtained with molecules targeting some of the above-mentioned pathogenic cascades, although it is sometimes difficult to systematize them into distinct classes, many of these molecules having multiple targets. Further, we will briefly discuss some more promising approaches, focusing mainly on the novel gene-silencing approaches.

The most commonly prescribed drugs for HD are medications to reduce chorea, namely tetrabenazine and deutetrabenazine. They both inhibit vesicular monoamine transporter type 2 (VMAT2) [108], thereby decreasing the amount of available dopamine and diminishing choreic movements by inhibiting dopaminergic signaling. Based on the results of two randomized controlled trials [75,77], tetrabenazine and deutetrabenazine are the only two FDA-approved drugs for the treatment of chorea in HD [89]. The side effects are not negligible. Tetrabenazine, possibly by depleting other monoamines, such as serotonin and norepinephrine, can cause depression, anxiety, and increase suicidal risk [109], while deutetrabenazine does not potentiate depression but also increases the risk of suicide [77] in patients already prone to psychiatric disturbances. The currently ongoing study, NCT02509793, aims to assess the effect of tetrabenazine-induced serotonin depletion on impulsivity and depression [109], while 2 other trials, NCT04301726 and NCT04713982, will analyze the effect of deutetrabenazine on dysphagia (NCT04301726) and on speech and gait [62]. Valbenazine, the most selective enantiomer for VMAT2, currently approved for the treatment of tardive dyskinesia [110], has been tested in a phase 3 clinical trial (KINECT-HD, NCT04102579) to evaluate its efficacy, safety, and tolerability in HD for the treatment of chorea, but the results have not been released yet [62].

Antioxidants have been actively pursued in many neurodegenerative diseases in which oxidative stress has been shown to be involved [22], as well as in ischemic stroke [34]. However, due to ROS being involved in complex pathogenic cascades, the one target-one drug approach has been marked by many failures. Dietary antioxidants have many beneficial actions and would be more likely to succeed [111], but they have poor bioavailability and must be engineered to overcome this drawback. Resveratrol is a nonflavonoid polyphenol that acts in various neurodegenerative diseases by activating metabolic sensor/effector proteins, including AMP-activated protein kinase (AMPK), sirtuin 1 (SIRT1), and PGC-1α [60]. Sirtuins (SIRT 1–7) are a group of NAD^+^ (nicotinamide adenine dinucleotide)-dependent lysine deacetylases, which can initiate a series of adaptive responses and regulate the metabolic efficiency of the cell [112]. In vitro studies have shown that resveratrol promotes SIRT1 deacetylase activity [113], while in transgenic mouse models of HD, modest increases in PGC-1α and Nrf-1 mRNA expression were achieved with resveratrol preparations [61]. Other dietary antioxidants, such as polyphenols, showed promising results in chemical rat HD models: quercetin restored the activities of catalase and superoxide dismutase 1 [114], while curcumin exhibited free radical scavenging properties in the same model [115]. Another free radical scavenger, Edaravone, reduced the markers of oxidative stress in a quinolinic acid rat HD model [116], while alpha-lipoic acid improved survival in transgenic mouse models [117].

Another promising strategy, in our opinion, is improving the availability of BDNF in the striatum and providing trophic support for the MSNs. Although BDNF can be increased with dietary interventions [118] or physical exercise [119], various pharmacological strategies, genetic approaches, or direct infusions of BDNF have also been evaluated in HD models [89]. Riluzole, an NMDAR inhibitor, increased BDNF expression and led to improved HD symptoms in transgenic HD mice [120] and even in human patients [121]. Selective serotonin reuptake inhibitors are also able to increase cerebral BDNF levels, with promising results obtained with paroxetine [91], fluoxetine [92], sertraline [93], and amitriptyline [94] in mice. Intranasal administration of human recombinant BDNF improved depressive-like behavior in transgenic mice [97], while intranasal administration of neuropeptide Y led to increased expression of BDNF and reduced HD neuropathology in R6/2 mice [98]. Another molecule, PACAP38 (pituitary adenylate cyclase-activating polypeptide 38), also delivered intranasally, enhanced synaptic plasticity and attenuated memory deficits in R6/1 mice, outcomes at least in part attributable to increased BDNF expression [99]. Delivering the *BDNF* gene using viral vectors is an attractive approach, allowing constant production of the protein [89]. Intraventricular injections of adenoviral BDNF resulted in increased neurogenesis and differentiation of the neurons into MSNs [122], while adenovirus-mediated induction of BDNF in astrocytes led to delayed onset of motor impairment in R6/2 mice [123]. However, several issues regarding the regulation of the amount of BDNF produced, the vector-associated inflammation, and the risk of tumorigenesis caused by viral vector-mediated accidental mutagenesis must be settled before the approach can be translated into clinical practice [89,124]. The direct delivery of BDNF by implanting BDNF minipumps, although beneficial in animal models, is hardly feasible in human trials [125], but the striatal transplantation of cells engineered to express stable levels of BDNF proved promising in animal models [126]. To overcome the risk of graft rejection, researchers are striving to develop non-tumorigenic human neural stem cells which would express BDNF [127].

### 3.2. Therapeutic Strategies Targeting Mutant Huntingtin

Because HD is caused by an identified genetic mutation in the *HTT* gene encoding for Htt protein, targeting *HTT* transcription and its mRNA translocation has been intensely investigated in recent years. This direction has been boosted by the FDA approval of nusinersen, an antisense oligonucleotide administered intrathecally to treat spinal muscular atrophy (SMA) by modulating gene expression and increasing the production of survival motor neuron (SMN) protein [128]. Nonetheless, there are significant differences between the 2 inherited diseases because, in SMA, the need is to restore the function of a missing protein, while in HD, we need to reduce the function of a toxic protein [129]. Furthermore, given the essential role of Htt in embryonic development and survival of neurons [130], the complete deactivation of the *HTT* gene would not be desirable.

The therapeutic strategies which target mHtt production can be divided into two major classes: drugs that interact with the HTT gene, such as antisense oligonucleotides (ASOs) or RNA interference (RNAi) compounds, which accelerate the degradation of the transcript, and small molecules which alter mRNA splicing [131]; and agents that directly interact with the DNA, such as zinc finger transcriptional repressors (ZFTRs) and CRISPR/Cas9 (clustered regularly interspaced short palindromic repeats/CRISPR-associated protein 9)-based tools for genetic editing [132]

#### 3.2.1. Targeting the RNA

A. Antisense oligonucleotides

ASOs are synthetic single-stranded DNA sequences composed of a phosphate backbone and sugar rings connected to one of four bases [133]. The DNA sequence is complementary to the messenger RNA target, which binds pre-mRNA in the nucleus through Watson–Crick base pairing and targets the mRNA sequence for degradation by RNase H endonuclease [133], thereby reducing the target gene translation. They were first used by Stephenson and Zamecnik to inhibit viral RNA translation in Rous sarcoma [134].

DNA replication in a dividing cell requires a short strand of complementary nucleotides, called the RNA primer, to induce the activity of DNA polymerase. After DNA replication, RNase H recognizes the DNA/RNA complex and degrades the RNA primer. ASOs act in the nucleus and bind to their target RNA, mimicking the DNA/RNA complex, and recruit RNase H to degrade the complex [135]. Unfortunately, they have poor bioavailability, the blood-brain barrier preventing their entrance into the CNS, and are rapidly degraded by exo- and endonucleases in addition to having several off-target effects. Modifying the phosphate backbone or the sugar rings can increase the ASO binding to plasma proteins and its resistance to degradation by nucleases and maintain stable serum concentrations. To overcome these issues, the ASO backbone and sugar moieties can be modified, leading to improved pharmacologic properties and stability [136]. The modifications of the phosphate backbone include the replacement of the non-bridging oxygen atom with a sulphur atom in the phosphate groups, leading to a phosphorothioate (PS), replacing 3′-oxygen with a 3′-amino group, leading to phosphoamidate (PA), or replacing the charged phosphodiester linkage with a non-charged phosphoroamidate linkage and the sugar-phosphate backbone with a morpholine ring, leading to a phosphorodiamidate morpholino oligomer (PMO). Through complete replacement of the deoxyribose phosphate backbone with polyamide linkages, peptide nucleic acids, with increased biological stability but low solubility and cellular uptake, have been obtained [137]. The sugar moiety has been modified at the 2′-position, obtaining 2′-O-methyl (2′-OMe) and 2′-O-methoxy-ethyl (2′-MOE) ASOs, or the 4′-carbon can be linked to the 2′-methyl group, leading to a locked nucleic acid (LNA). Another sugar modification is constraining the 2′-residue to the 4′-position of the sugar ring, obtaining an S-constrained-ethyl (cET) ASO [138]. Each of these modifications has characteristic advantages. Table 3 provides a summary of the available ASOs.

**Figure 2:**
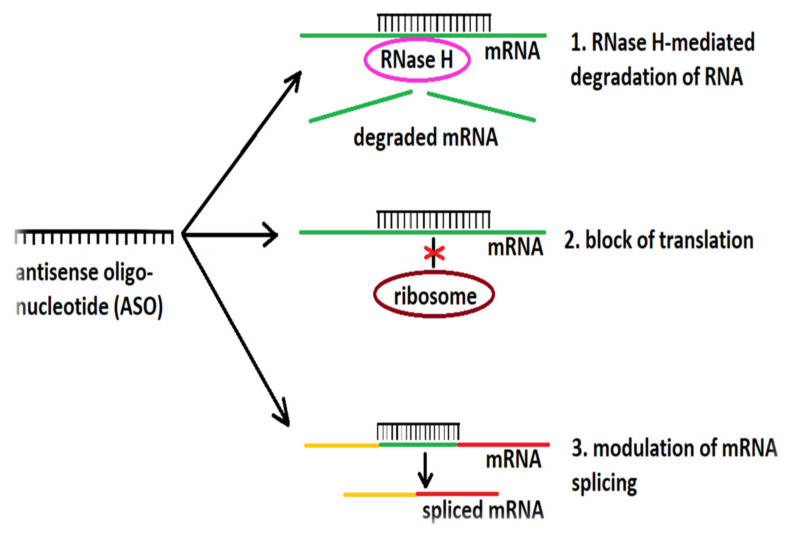
Mechanism of action of ASOs. ASOs influence gene expression through three principal mechanisms: (1) recruitment of RNase H and degradation of mRNA, (2) steric block of ribosome binding, and (3) modulation of mRNA splicing.

Unfortunately, replacement of the phosphate backbone with a phosphorothioate linker triggers RNase H-mediated degradation of the mRNA but also can trigger the immune system [135]. Phosphorodiamidate morpholinos (for example, eteplirsen, approved in September of 2016 by the FDA for the treatment of Duchenne muscular dystrophy but rejected in 2018 by the European Medicines Agency) [146,147] or 2′-O-methyl ASOs (such as nusinersen, used for the treatment of spinal muscular atrophy) [148] have modified ribose sugars, which increase the ASO’s resistance to degradation and enhance target specificity [131]. Alternatively, some ASOs, such as morpholino ASOs, do not recruit RNase but bind to the target RNA and prevent ribosomal attachment, while others modify RNA splicing. Genes comprise a promoter region (to which RNA polymerase binds) and several coding segments (exons) separated by non-coding segments (introns) [131]. DNA transcription results in a complementary RNA molecule, the pre-mRNA, containing both exons and introns. Specific nucleotide sequences placed in and around the exon/intron boundaries recruit splicing factors, which remove the introns and produce mRNA molecules that contain only exons and will undergo ribosomal translation. Certain ASOs can bind to these sequences and alter pre-mRNA splicing [131]. Figure 2 provides a schematic overview of the mechanism of action of ASOs.

Single-stranded DNA diffuses in the CNS, being taken up by neurons and glial cells. Thereby, administration into the CSF (intrathecal administration in large mammals or intraventricular delivery in mice) results in the drug being delivered to the brain [140,149]. In HD, reduction of HTT mRNA leads to a reduced expression of mHtt and improvement of neurological deficits and can even postpone onset if achieved in presymptomatic animals which carry the mutation [129].

However, given the size and complexity of the human brain compared to animal brains, the ability of ASOs to diffuse may vary considerably. Furthermore, off-target effects, leading to hepatotoxicity, or recognition of ASOs by toll-like receptors on immune cells and triggering immune responses leading to thrombocytopenia, raise certain concerns [150].

Nonetheless, several ASOs are being investigated in HD models, and an ASO targeting human huntingtin, IONIS-HTT_RX_ (tominersen), has entered human clinical trials (NCT02519036—Safety, tolerability, pharmacokinetics, and pharmacodynamics of IONIS-HTT_RX_ in patients with early manifest Huntington’s disease) [69]. The compound developed by Hoffman-La Roche is a 20-nucleotide phosphothioate sequence with 2’-O-methoxy ethyl modifications at each end of the DNA-like central region to improve cellular distribution and RNase activation [129]. In animal models, the compound gained wide distribution in neurons and glia, leading to reduced brain atrophy in the R6/2 model [149]. The pathological benefits of a bolus ASO treatment were shown to outlast the presence of the drug, suggesting that a brief stop in mHtt synthesis can help cells repair—a phenomenon called the “huntingtin holiday” [151]. In the clinical trial, there was a dose-dependent reduction of mHtt in the cerebrospinal fluid, although not necessarily reflecting the reduction of cellular mHtt, and no serious adverse events [152]. A randomized open-label extension trial (NCT 03342053) continued the assessment of adverse effects. It concluded in 2019, but the results have not yet been published [153]. Another randomized, double-blinded, phase 3 clinical trial aimed at evaluating the efficiency of tominersen (GENERATION HD1 (NCT03761849). Although it was expected to be finished in 2022, it was discontinued due to concerns regarding the benefit-to-risk ratio raised by the review of data by an independent data monitoring Committee, as was the open-label extension phase, GEN-EXTEND, NCT03842969 [153,154]. Because tominersen is non-allele-selective, it reduces the expression of both normal and mutant huntingtin, a situation in which long-term use in symptom-free carriers of the genetic defect may raise safety concerns. In addition, polyCAG stretches are contained in numerous human genes, mainly encoding for transcription factors. As such, non-allele-specific ASOs can downregulate other genes as well, leading to off-target adverse effects [129].

An alternative strategy is to target single nucleotide polymorphisms (SNPs) that are present on a limited number of HD haplotypes and which would allow for specific silencing of the mutant gene. The huntingtin gene has many SNPs, with sequencing techniques being able to establish which allele a SNP is located upon [155,156]. Certain SNPs accompany CAG mutations more frequently. It is estimated that drugs targeting the three most common SNPs could treat roughly 80% of patients with HD of European ancestry [157]. A 2’-O-methyl (2’OMe) modified RNA with a phosphorothioate backbone carrying 7 consecutive CUG nucleotides reduced the mHtt mRNA by 83% and the wild-type transcript by only 43%, suggesting that the inhibition by (CUG)7 depends on CAG repeat length [156] and led to clinical improvements in R6/2 mice [158]. Two allele-specific ASOs have been developed, targeting the rs362037 (SNP1) and the rs362331 (SNP2) single nucleotide polymorphisms [159], which entered clinical trials (PRECISION-HD1 and PRECISION-HD2; NCT03225833, NCT03225846) [62,159] evaluating their tolerability, pharmacokinetics, pharmacodynamics, and safety. Although preliminary data from the PRECISION-HD2 study suggested a dose-dependent reduction in mHtt, in 2021, both trials were stopped by the sponsor due to a lack of significant change in mHtt levels, as were the 2 open-label extension studies (NCT04617847 and NCT04617860) [160]. Another allele-specific ASO, targeting an undisclosed SNP (SNP3), showed promising results in preclinical studies [161], leading the manufacturing company to submit an application for a clinical trial in 2020 [153].

Other allele-specific ASOs, which are tested in preclinical trials, target the expanded CAG repeats in mHTT mRNA (CUG7) or SNPs associated with HD alleles [162], while others modulate the splicing of pr-mRNA, inducing the skipping of exon 12 [163], resulting in a 586 amino acid N-terminal fragment in rodents, which does not express the cleavage sites. Peptide-conjugated ASOs, administered systemically, showed broad CNS distribution and efficacy in spinal muscular atrophy mouse models, being also pursued for the treatment of HD [164]. Finally, some ASOs do not target mHtt, but genes involved in the DNA damage response pathway because defective repair of double-streak breaks in the DNA, the most lethal forms of DNA injury, have been found in postmortem examinations of brain cells in HD patients [165]. Mutant Htt impairs the non-homologous end-joining (NHEJ)-mediated DNA DSB repair mechanism in neurons [166,167], suggesting that alterations of the DNA repair mechanism could have important contributions to the prominent cell loss. Targeting the mutS homolog 3 (MSH3) with TTX-3360 with ASOs, developed by Triplet Therapeutics, proved safe and efficient in mouse models of HD [153].

B. RNA interference approaches

RNA interference (RNAi) molecules can induce gene silencing either through direct sequence-specific cleavage of perfectly complementary RNAs or through translational repression and transcript degradation for imperfectly complementary structures [168]. Depending on sequence and structure, these molecules can be divided into short interfering RNAs (siRNAs), short hairpin RNAs (shRNAs), and microRNAs (miRNAs) [129].

Long double-stranded RNA (dsRNA), such as viral genetic material, is processed to short interfering RNAs (siRNAs), which mediate the RNAi response (at least in plants, nematodes and insects), by a dsRNA-specific endonuclease (RNase III) called Dicer, that acts together with TRBP (trans-activation response (TAR) RNA binding protein) and protein activator of protein kinase PKR (PACT) in a complex. siRNAs are loaded into a multiprotein complex, the RNA-induced silencing complex (RISC), where Argonaute 2 (AGO2) cleaves the passenger (sense) strand of the siRNA and generates a single-stranded antisense strand which guides RISC to complementary sequences in target mRNAs. These target mRNAs are cleaved between bases 10 and 11 relative to the 5′ end of the siRNA guide strand, followed by degradation of the cleaved mRNA fragment by cellular exonucleases [169]. After being activated by the siRNA guide strand, RISC can undergo multiple rounds of mRNA cleavage, resulting in a significant post-transcriptional gene silencing (PTGS) response [168]. Thus, mRNA translation and protein synthesis are inhibited.

**Figure 3:**
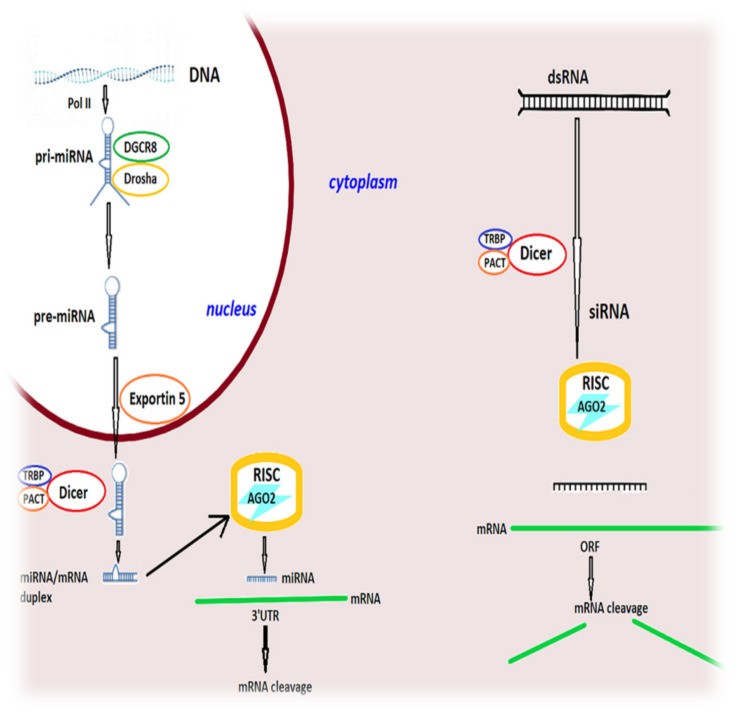
Mechanisms of RNA interference in mammalian cells. Endogenously encoded primary microRNA transcripts (pri-miRNAs) result from the activity of RNA polymerase II (Pol II) and are processed by Drosha–DGCR8 (DiGeorge syndrome critical region gene 8), generating precursor miRNAs (pre-miRNAs), which are exported into the cytoplasm by exportin 5. In the cytoplasm, pre-miRNAs are processed by the Dicer–TRBP–PACT complex and loaded into RISC and AGO2. The resultant mature miRNA recognizes target sites in the 3′ untranslated region (3′ UTR) of mRNAs and direct translational inhibition and mRNA degradation. In the cytoplasm, double-stranded RNAs (dsRNAs) are also processed by the Dicer–TRBP–PACT complex into small interfering RNAs (siRNAs), which are loaded into RISC and AGO2. The siRNA guide strand recognizes target sites to direct mRNA cleavage performed by the catalytic domain of AGO2. TRBP—TAR RNA-binding protein; PACT—protein activator of protein kinase PKR; RISC—RNA-induced silencing complex; AGO2—Argonaute 2; ORF—open reading frame.

MicroRNAs (miRNAs) are endogenous small RNAs that induce PTGS through translational repression in case of partial sequence complementarity with target mRNAs. However, in case of complete sequence complementarity between the miRNA and the target mRNA, the mRNA transcript will be cleaved by RISC [168]. Long primary miRNA transcripts (pri-miRNAs) are generated mainly by polymerase II and are trimmed by Drosha (RNase III), which functions together with the dsRNA-binding protein DGCR8 (DiGeorge syndrome critical region gene 8), into precursor miRNAs (pre-miRNAs). These pre-miRNAs are translocated by exportin 5 into the cytoplasm, where Dicer and its protein partners TRBP40 and PACT further process the pre-miRNAs into mature miRNAs and direct them into RISC [169,170]. Here, the RNA duplex associates with one of four Argonaute proteins (AGO1–4), and the antisense (guide) RNA strand will bind to target mRNAs with which it shares partial sequence complementarity, leading to mRNA degradation, translational repression and gene silencing, while the passenger strand is discarded [168,171]. Figure 3 provides a schematic representation of the mechanism of RNA interference in mammalian cells.

Short hairpin RNAs (shRNAs) are synthetic molecules with a short hairpin secondary structure delivered on a DNA plasmid. After transcription, the molecule mimics pri-miRNAs and is processed by Drosha to pre-shRNA, further exported from the nucleus by exportin 5 and acting as a substrate for Dicer, which targets it to RISC. Here the passenger strand is disposed of, and the antisense strand will lead to the degradation of the target mRNA [172]. Compared to siRNAs, which only transiently silence gene expression because their intracellular concentrations decrease following successive cellular divisions, shRNAs mediate long-term silencing of their target transcripts (over months or years), especially if polymerase II and III promoters are associated with driving expression of the shRNAs [168,172]. Table 4 provides a comparison of the advantages and disadvantages of siRNA and shRNA approaches.

However, if single-stranded DNA shows good CNS distribution after intraventricular or intrathecal administration, being taken up by neurons and glial cells, dsRNA is a negatively charged polymer and has limited diffusion, requiring special delivery methods, such as viral vectors, adeno-associated viral vectors, or nanotechnology approaches to ensure optimal effects and must be delivered directly into the brain [129]. Unprotected siRNAs can also be degraded by serum RNases. Several chemical modifications, such as including an O-methylpurine or fluoropyrimidine at the 2′ position of the ribose, can increase siRNA stability [168]. Further, conjugation of a cholesterol group to the 3′ hydroxyl group of the siRNA or packaging the compound into a liposomal particle or a specialized lipid bilayer known as a stable nucleic acid-lipid particle (SNALP) allows for intravenous delivery [173,174]. Coupling the therapeutic siRNA to antibody fragments or aptamers (single-stranded oligonucleotides which bind to specific proteins) or coating the nanoparticles with receptor-targeting ligands allow for cell-specific systemic delivery and lower dosage of the siRNA [168].

Other issues relate to the possibility of inducing an immune response, of saturation of the cellular RNAi machinery, and the induction of off-target effects [169]. Especially when using shRNAs, the passenger strand is on occasion retained and may induce unwanted inhibition of off-target mRNAs. By using modified, guide-only effectors based on miRNA miR451, this side effect can be overcome because miR451uses a Dicer-independent cleavage pathway, being processed by AGO2 in the cytoplasm and further cleaved by PARN (poly(A)-specific ribonuclease). Being AGO1, AGO3, and AGO4-independent, miR451-like effectors show decreased competition for endogenous miRNAs and have enhanced safety and reduced potential for off-targeting [175]. Combining AGO2-dependent shRNAs with a miRNA backbone enables tissue-specific expression and increases the safety of the therapeutic shRNA [176].

AMT-130 is the only gene therapy currently in clinical trials for HD [177]. It contains a gene encoding for a miRNA, which is administered as an intrastriatal injection, delivering the genetic material via adeno-associated virus vector serotype 5 (AAV5) [178]. In animal models and HD patient-derived cultured neurons, AMT-130 induced a robust and sustained reduction in mHtt protein and mRNA levels [153,179,180] and was shown to be safe in non-human primate HD models [181]. It is currently tested in a phase 1b/2 clinical trial on 26 HD patients (NCT04120493), which continues with an open-label extension phase trial (NCT05243017), expecting to enroll 15 patients for safety, tolerability and efficacy. The trials are expected to be completed in 2026 and 2027, respectively [62].

Other RNAi therapies tested in preclinical trials are VY-HTT01, a miRNA delivered via adeno-associated virus vector serotype 1 (AAV1) and leading to mHtt mRNA degradation, which showed a significant reduction in mHtt protein, as well as motor and behavioral improvement in mice [182], and an AAV1-delivered shRNA, which also showed good preclinical results [153].

In addition, small, orally administered molecules which modify pre-mRNA to include or exclude target exons (splice modulation) are also valid approaches, although their systemic distribution and lack of specificity may increase the risk of adverse effects [183]. PTC518 is already tested in a phase 2a clinical trial on 162 participants (NCT 05358717) for safety and efficacy, while LMI070 (branaplam), initially developed for spinal muscular atrophy but subsequently found to reduce mHtt protein, entered a phase 2 clinical trial aiming to enroll 75 participants (NCT05111249, VIBRANT-HD) to evaluate safety and efficacy and find the correct dose [62]. An RNA splice modulator targeting Spt4, a transcription elongation cofactor required for expanded CAG repeat transcription, is another molecule with promising results in preclinical trials, but no clinical trial has yet begun [62].

#### 3.2.2. Targeting the DNA

Although targeting DNA could improve all aspects of HD and could raise the prospect of “editing” the DNA in the patients’ germ line to benefit future offspring, it carries several ethical issues [184]. Still, two DNA-targeting gene therapies are currently under investigation: zinc finger proteins (ZFPs) and CRISPR/Cas9 [129]. Both use viral vector-mediated, intracranially injected protein-coding sequences which transduce cells and lead to the production of functional proteins.

A. Zinc finger proteins

Zinc fingers are naturally occurring structures that bind specific DNA sequences. Synthetically generated zinc finger proteins contain a zinc finger array directed to a DNA sequence (one finger per three bases) combined with a functional domain that acts on the DNA [129]. They can be subdivided into zinc finger nucleases, which cleave the DNA, and zinc finger transcription factor repressors (ZFTRs), which modulate gene expression [185]. The use of zinc finger nucleases, although able to “edit” target genes, is currently not sufficiently precise or predictable to be used as therapy in postmitotic patient cells, especially for polyCAG stretches [186]. ZFTRs act by binding the zinc finger array to a sequence near the 3′ end of the DNA sense strand, thereby bringing the transcriptional repressor close to the promoter of the target gene. Fortunately, the promoter sequence of the *HTT* gene is closer to the 3′ end than in other polyCAG-containing genes [187], which allows for ZFTR selectivity for *HTT* over other genes as well as for the mutant gene over the wild-type allele [129] and leads to decreased mHtt levels without altering the gene itself [183].

Currently, there are 2 ZFTRs in preclinical evaluation: TAK-686 and ZF-KOX1 [153,188]. Both have shown promising results in animal models, with reduced mHtt expression and behavioral improvements [129,189]. However, zinc finger proteins can trigger immune reactions due to the production of non-native proteins and lead to neuronal death. To overcome this issue, Agustin-Pavón and colleagues used a polyCAG-targeting ZFTR combined with a non-viral promoter and a repressor element designed to be homologous with the host protein (mouse protein), achieving more sustained gene silencing [188].

B. CRISP/Cas therapies

Clustered regularly interspaced short palindromic repeats (CRISPR) and the CRISP-associated system (Cas) enable the immune system to recognize and destroy foreign DNA. All CRISPR therapies are delivered via viral vectors and intracranial injection [190]. Cas9 (CRISPR-associated protein 9), a nuclease, has been extensively used as a genome editing tool in the past decade [153]. Cas9 is combined with a synthetic guide RNA and binds to its target DNA sequence, inducing double-strand breaks, which are repaired by error-prone non-homologous end joining, leading to frameshifts that can impair gene expression [191]. In HD, CRISPR/Cas9 strategies may correct HD alleles by removing expanded CAG repeats, inactivate HD-associated alleles, or target the HTT gene itself [192,193].

The approach has been tested in cell lines, where it excised expanded CAG repeats in exon 1 of the HTT gene [191,194], as well as in animal models, where it reduced mHTT expression, astrocyte reactivity, and led to improvement of motor symptoms [195,196]. However, concerns about off-target mutations caused by CRISP/Cas9 gene editing have been raised, which need to be solved before the technology could enter clinical trials [197].

C. TALEN therapies

Transcription activator-like effector nucleases (TALENs) have domains containing repeating peptides which bind to DNA nucleotides and cause double-strand breaks through the artificial nucleases, thereby correcting or deleting specific segments [153]. They are still in the discovery stages, with no established effective delivery systems, and no valid information regarding toxicity or impact on phenotype is available to date. Nonetheless, in yeast cells, TALENs efficiently removed expanded CAG repeats, while in HD patient-derived fibroblasts, the transcription of the mHTT gene was prevented, while wild-type HTT gene expression was unaltered by using a TALEN and SNP-specific transcription activator-like effector (TALE-SNP) [198].

### 3.3. Therapeutic Strategies Targeting Cell Loss

Neuronal loss due to HD pathology could be replaced by using stem cell therapies, which could also provide pro-survival factors and improve regeneration. However, there is the possibility of rejection or of inducing immune reactions [199].

In the most advanced stage among stem cell-based therapeutic approaches is an allogenic mesenchymal therapy using human immature dental pulp stem cells developed by the Brazilian company Cellavita [153]. In a phase 1 clinical trial (NCT02728115/SAVE-DH), 6 HD patients received 3 doses of 1 or 2 million cells/kg body weight intravenously, each dose 30 days apart, and were followed for 5 years looking for safety, tolerability, and efficiency [62]. Preliminary results reported after 2 years of follow-up raised no concerns [200] and justified the application for a phase 2 clinical trial (NCT03252535—Dose-response Evaluation of the Cellavita HD Product in Patients with Huntington’s Disease (ADORE-HD), which began in 2018 and enrolled 35 participants who received 3 cycles of monthly intravenous delivery of 1 or 2 million cells for 3 months, the cycles being repeated after 120 days [62]. Its primary aim is to identify the optimal dose for the best clinical response. The study was completed in March 2021, but no results have been published so far. However, an open-label extension phase 2/3 trial is registered (NCT04219241—ADORE-EXT) but not yet recruiting [62].

Other mesenchymal stem cell (MSC) approaches are in preclinical trials. Autologous mesenchymal cells originate in the umbilical cord, bone marrow, or adipose tissue and can differentiate into many cell types, including neural cells [201], being self-renewing and able to release neurotrophic factors [153], without the risk of teratoma. Umbilical cord-derived MSC injected into the striatum of R6/2 mice reduced the neuropathological changes but did not lead to motor improvements [202], while striatal transplantation of umbilical cord-derived MSC in rats resulted in reduced striatal atrophy, diminished oxidative stress, and enhanced cell viability in a 3-nitropropionic acid (3NP) rat model of HD [203]. A single-center phase 1/2 clinical trial with bone marrow-derived autologous mononuclear cells injected intrathecally to patients with HD was initiated in 2014 (NCT01834053) and estimated to be completed in 2016, but its status and results are currently unknown [62].

Induced pluripotent stem cell-derived neural stem cells (iPS-NSCs) are reprogrammed somatic adult cells able to differentiate into various cell types [204]. Mouse iPS-NSCs injected into the striatum of 3NP rat HD models resulted in differentiation of the iPS-NSCs into striatal neurons and glia, with partial recovery of the striatal volume and improved learning and memory [205], while human iPS-NSCs transplanted to the striatum reduced inflammation, replaced some neural cells and promoted neurogenesis in a quinolinic acid-induced rat HD model [206]. Healthy mice iPS-NSCs injected into the striatum of YAC128 HD mice led to differentiation of the stem cells into neural cells, followed by increased striatal BDNF and motor improvement [207].

Fetal stem cell therapy replacement therapy started to be investigated more than 20 years ago [153]. Early studies showed the approach to be safe but lacking long-term efficacy [208]. However, the need for immunosuppressants to mitigate graft rejection diminishes compliance and has raised safety concerns [209], although some researchers have shown that, due to the unpredictable nature of the patients’ immune response after the graft, immunosuppressants should not be used excessively [210]. A single-center, phase 1 open-label clinical trial (ISRCTN52651778) to evaluate the safety and efficacy of fetal stem cell transplant in HD was started in 2018 and is expected to be completed in 2023 [177,211]. Ethical concerns regarding the use of fetal cells also persist [153], and the reduced vascularization and diminished astrocyte and pericyte numbers following grafting discard the approach as a permanent cure [212].

Autologous iPSCs would eliminate or minimize the possibility of rejection, but the genetic defect would have to be corrected prior to the cell therapy. Genome editing by ZFN or CRISPR/Cas9 still raises concerns regarding the non-specific targeting of the mHTT allele and silencing of the normal HTT gene, the long-term effects of which have yet to be determined. A relatively recent study reported that the elimination of Htt in adult mice resulted in motor and behavioral decline [213]. Gene silencing of the mutant gene using shRNAs is a more validated therapeutic option. In a transgenic mouse model of HD, autologous iPSCs with reduced mHtt levels by stable expression of shRNA grafted into the striatum differentiated into neurons and glia, leading to significant improvements in motor function and increased life span [214]. A clinical trial evaluating the safety and efficacy of autologous stromal stem cells in various neurodegenerative diseases (NCT03297177), sponsored by Regeneris Medical Inc., was planned to start in January 2020 but is not yet recruiting [62].

### 3.4. Strategies Targeting Neuroinflammation

Neuroinflammation has been increasingly shown to contribute to neuronal damage in Alzheimer’s disease [215], Parkinson’s disease [216], amyotrophic lateral sclerosis [40], stroke [41], as well as in Huntington’s disease [217]. Expression and accumulation of mHtt in neurons, as well as in microglia, have been implicated in microglial activation and ignition of the neuroinflammatory cascade [218]. As such, therapies trying to mitigate inflammatory responses have been developed and evaluated.

#### 3.4.1. Laquinimod

Laquinimod is an orally administered small immunomodulatory molecule which shifts T helper cell (Th) polarization towards a Th2 polarization and promotes BDNF production [219]. It is currently used in the treatment of relapsing-remitting multiple sclerosis [220] but has also been shown to reduce Bax expression and caspase-6 activation in cultured neurons [221], as well as to improve striatal pathology and motor function in R6/2 HD mice [95]. However, in a clinical trial on 325 patients (LEGATO-HD, NCT02215616), although it reduced caudal atrophy, laquinimod failed to improve the functional status of the enrolled patients [222].

#### 3.4.2. Drugs Targeting TNF-α

Tumor necrosis factor-α (TNF-α) is a cytokine associated with immune response, inflammation, and apoptosis [12,41]. Increased levels of TNF-α have been found in the serum, brain tissue, and cerebrospinal fluid of HD patients as well as of HD gene carriers [223]. Despite encouraging results obtained with an engineered inactive form of TNF-α injected intracerebroventricularly in R6/2 transgenic mice [224], systemic delivery of etanercept, a TNF-α-inhibiting drug, failed to improve cognitive and motor deficits in R6/2 mice and only partially reduced brain atrophy [225], an effect possibly related to the poor blood-brain barrier penetrance of the drug.

#### 3.4.3. Antibody-Based Therapies

Antibody-based therapies have been evaluated in synucleinopathies and tauopathies [18] and are emerging as potential therapies in genetic disorders of the central nervous system as well [153].

ANX005 is a monoclonal antibody that prevents the complement cascade activation, shown to lead to neuroinflammation, neurodegeneration and synapse loss by targeting C1q [226]. In a randomized, double-blind, ascending dose, phase 1 clinical trial in healthy volunteers (NCT03010046), ANX005 proved to be safe [62], which led the developing company to initiate phase 2 studies in Guillain–Barré syndrome, amyotrophic lateral sclerosis, and a form of autoimmune hemolytic anemia [153], as well as in Huntington’s disease (NCT04514367) to evaluate the safety, pharmacokinetics and pharmacodynamics of 7 intravenous infusions of ANX005 in 28 participants. The trial is estimated to be completed in July 2022 [62].

VX15/2503 (pepinemab) is an IgG4 monoclonal antibody that inhibits semaphorin 4D, a protein that promotes glial cell activation and leads to oligodendrocyte and neural precursor cell apoptosis [227]. In YAC128 mice, the compound reduced cortical and striatal atrophy and improved cognitive symptoms, although it did not influence motor ones [228]. In a phase 2, double-blinded clinical trial conducted on patients with late prodromal or early manifest HD to evaluate safety, pharmacokinetics and pharmacodynamics (SIGNAL, NCT02481674), VX15/2503 appeared safe and even efficient in prodromal HD, although it did not alter motor, behavioral, or cognitive function in the established disease [153].

Antibodies targeting mHtt can be delivered in a simpler form, as intrabodies via viral vectors, and can bind to various epitopes on a target protein, inactivate proteins, stop the intracellular misfolding of the protein, or enhance protein clearance [153]. In HD, their target is the polyP/proline-rich region and N-terminal exon 1 domain [229]. rAAV6-INT41 is an intrabody targeting the polyP/proline-rich region, which, combined with a viral vector and injected into the striatum of R6/2 mice, reduced small and large mHtt aggregates and improved cognitive deficits [230]. Intrabodies targeting mHtt aggregates have also shown beneficial results but raised concerns regarding the possibility of increasing aggregation [229]. One such intrabody is W20, which was tested successfully in a mouse model of HD, where it reduced the accumulation of aggregates in the striatum and cortex, diminished the levels of reactive oxygen species and proinflammatory cytokines, and improved motor performances and memory [229]. Antibodies have the potential to target extracellular mHtt [231], but the possible benefits of active immunization are not yet known, and questions regarding safety persist.

Passive immunization with monoclonal antibodies, such as C16–17, has shown positive results in vitro and in a mouse model of HD [232]. However, antibody therapies could work best together with RNA-based treatments in order to target intracellular as well as extracellular mHtt [231].

Table 5 summarizes these novel therapeutic approaches in Huntington’s disease.

**Figure 4:**
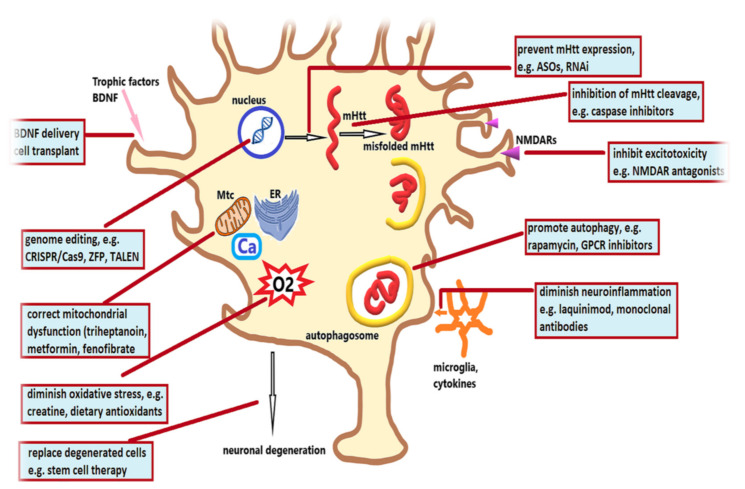
After much research has focused on elucidating the intricate pathogenic cascades leading to neuronal degeneration in Huntington’s disease, therapeutic strategies have targeted various aspects of these mechanisms: the defect gene could be corrected with CRISPR/Cas9, zinc finger protein (ZFP) or TALEN (transcription activator-like effector nucleases) approaches; mutant huntingtin (mHtt) expression could be prevented with antisense oligonucleotides (ASOs) or RNA interference (RNAi) approaches; caspase inhibitors could reduce the abnormally folded mHtt aggregates; autophagy enhancers (rapamycin or G protein-coupled receptors—GPCRs) could promote mHtt clearance; attempts have been made to stabilize mitochondria (Mtc) and prevent mitochondrial dysfunction with metformin, fenofibrate, or triheptanoin; this could also reduce oxidative stress, which can be mitigated with dietary antioxidants (engineered for improved blood brain barrier penetrance) or creatine; stem cell therapies could replace degenerated cells, and additionally enhance the availability of brain derived neurotrophic factor (BDNF), as well as other neurotrophic factors; N-methyl-D-aspartate receptor (NMDAR) antagonists could reduce excitotoxic cell death, while neuroinflammation could be diminished with monoclonal antibodies or other anti-inflammatory agents, such as laquinimod.

Figure 4 summarizes the various therapeutic strategies evaluated in the treatment of HD according to their targets.

## 4. Conclusions

Despite the identification of the genetic defect leading to Huntington’s disease almost 30 years ago, currently available therapeutic options address only symptoms of the disease, with no significant progress over the past 20 years [233]. Perhaps the most important shortcoming of available therapies is their inability to act on specific targets, influencing only downstream processes and allowing the pathogenic cascades to progress where they are not inhibited [234].

The recent interest in therapies that target mHtt DNA and RNA opens new exciting opportunities and, combined with strategies targeting glutamatergic neurotransmission, BDNF signaling, or mitochondrial function, will probably result in better outcomes. In addition, targeting the gut microbiota, due to its alteration in HD (as in other neurodegenerative diseases) [235], could add to therapeutic efficiency [236].

However, the genetic therapeutic approaches are still in their infancy. Questions on the safety of non-selective mHtt lowering, the incidence of off-target effects in large populations, and the safety of CRISPR therapies are still unanswered [237]. Furthermore, the necessity for invasive administration could be a major drawback [129], limiting adherence and access to therapy.

Nonetheless, based on the encouraging latest developments, we might soon witness a new era in the treatment of HD.

## Figures and Tables

**Table 1:** Pathogenic cascades involved in Huntington’s disease.

Pathogenic Mechanism	Action of mHtt	Resulting Abnormalities	References
Excitotoxicity	Altered transcription of the GluN2B subunit of NMDARs Impaired interaction with PSD95 Increased cytosolic Ca^2+^ concentration	Abnormal sensitivity and distribution of NMDARs, favoring extrasynaptic NMDARs Activation of calpains and calcineurin, leading to apoptosis	[10,11] [15,16]
Impaired proteostasis	Sequestration of chaperones in mHtt aggregates Sequestration of Rhes into mHtt aggregates	Enhanced abnormal protein folding, overwhelming of the UPS Decreased autophagy	[17,18] [24]
Mitochondrial dysfunction	Direct interaction with Mfn2 Increased mitochondrial Drp1 translocation Reduced complex II, III, and IV activity Direct interaction with mitochondrial proteins Sequestration of GAPDH and HAP1 into mHtt aggregates Binding to the IP3R on the ER	Impaired mitochondrial fusion Increased mitochondrial fission Altered cellular energy supply Mitochondrial depolarization, opening of the MPTP, release of pro-apoptotic factors Impaired mitochondrial trafficking Ca2+ release from ER stores, increases in cytosolic Ca2+ concentration, loss of dendritic spines	[28] [28] [29] [12] [5] [32]
Oxidative stress	mHtt-induced mitochondrial dysfunction increases ROS production, leading to oxidative damage to proteins, lipids, and DNA	Mitochondrial calcium overload, opening of the MPT BER, OGG1 activity triggering further expansion of the CAG repeats in the HTT gene	[35] [37]
Transcriptional dysregulation	Impaired transcription of CREB, PGC-1α Nuclear translocation of REST Increased transcriptional activity of p53	Impaired synthesis of endogenous antioxidants Repression of BDNF gene transcription Upregulation of pro-apoptotic factors, such as BAX, PUMA	[36] [44,45] [46]
Reduced BDNF	Nuclear translocation of REST, accumulation of REST/NRSF in nuclei Sequestration of GAPDH, HAP1, and dynein into mHtt aggregates	Impaired BDNF synthesis Impaired BDNF transport from cortical neurons to the striatum	[44,45,48] [49]
Dysfunction of glial cells	Downregulates the expression of astrocytic Kir4.1 channel Binds to nMYRF in oligodendrocytes	Alteration of astrocytic membrane potential and sensitivity to neuromediators Myelination deficits	[52] [54]
Neuroinflammation	Promotes the expression of pro-inflammatory cytokines	Microglial M1 polarization Activation of the JAK/STAT and MAPK pathways	[41] [57]

**Table 2:** Overview of preclinical research and clinical trials with molecules targeting specific pathogenic cascades involved in Huntington’s disease.

Targeted Mechanism	Molecule	Effect	Trials	Results	References
mHtt aggregation	Epigallocatechin 3 gallate	Suppresses Htt aggregation	Clinical trial (NCT01357681, ETON trial)	Not published	[59]
	Resveratrol	Activates AMPK, SIRT1, increasing PGC-1α and Nrf2 mRNA expression	Mouse models Clinical trial (NCT02336633, REVHD)	Not posted	[60,61] [62]
	Rapamycin	Inhibits mTOR and activates autophagy, promoting mHtt aggregate clearance	Drosophila and mouse HD models, toxicity precludes human use	Decreased accumulation of mHtt aggregates, improved motor performance	[63]
mHtt cleavage	Minocycline	Inhibits caspase 1 and caspase 3	R6/2 mice Clinical trial (NCT00029874)	Improved phenotype Negative results	[64] [65]
	Nilotinib	Inhibits a tyrosine kinase involved in apoptosis, autophagy modulator	Clinical trial (NCT03764215, Tasigna-HD)	No published results	[62,66]
Excitotoxicity	Riluzole	Inhibits glutamate neurotransmission, enhances EAAT2 activity	Transgenic mice, primate HD model HD patients (NCT00277602)	Improved abnormal movements, reduced striatal atrophy, increasing survival Transient improvement of chorea	[67,68] [69,70]
	Memantine	Antagonizes extrasynaptic NMDARs	Chemical animal HD models Transgenic mouse models Memantine + risperidone in single HD patient	Reduced striatal neuronal loss Worsened outcome Slowed down progression of motor symptoms and cognitive decline	[71] [72,73]
	Lamotrigine	Glutamate antagonist	Chemical mouse models Clinical trial	Restored antioxidative defense mechanisms, improved behavior Null results	[1] [74]
	Tetrabenazine, Deutetrabenazine, Valbenazine	Inhibits vesicular monoamine transporter type 2 (VMAT2), decreases dopamine in the striatum	HD patients (NCT01451463; NCT00219804) (NCT01795859) HD patients (NCT04102579, KINECT-HD), NCT04400331	Diminished chorea No published results	[75,76] [62,77,78]
	Dextromethorphan	NMDAR antagonist	Clinical trial (NCT03854019), in combination with quinidine	No results released	[62]
Mitochondrial dysfunction and oxidative stress	Creatine	Stimulates mitochondrial respiration	R6/2 mouse models HD patients (NCT00026988) NCT01412151, CREST-X NCT00712426, CREST-E	Neuroprotective, slowed down the development of neuropathology Reduced markers of oxidative DNA damage No clinical benefit No clinical benefit	[79] [80] [62,68] [81,82]
	Coenzyme Q10	Interacts with ROS, improves ATP production	HD patients, NCT00608881, 2CARE	No clinical benefit	[83]
	Eicosapentaenoic acid (EPA)	Binds to mitochondrial PPAR, inhibits caspases, downregulates the JNK pathway	HD patients, NCT00146211, TREND-HD	No clinical benefit	[84,85,86]
	Metformin	Activates AMPK	In vitro and fly models R6/2 mice HD patients (NCT048266920, Test-ing METformin)	Neuroprotective effect Increased lifespan Ongoing, assessing its effect on cognitive decline	[87] [88] [62,89]
	Fenofibrate	Activates PGC-1α, promotes mitochondrial biogenesis	HD patients (NCT03515213)	ongoing	[62]
	Triheptanoin	Increases acetyl-CoA, a substrate of the Krebs cycle	HD patients, NCT02453061, TRIHEP3	No results released	[62,90]
Increase in BDNF	Selective serotonin reuptake inhibition (paroxetine, fluoxetine, sertraline, amitriptyline)	Activates the MAPK/ERK signaling pathway, BDNF/tyrosine kinase B pathway	Mouse models	Improved HD symptoms	[91,92,93,94]
	Immunomodulators: laquinimod, glatiramer acetate	Reduces NF-κB activation, upregulates BDNF	Mouse models	Improved mitochondrial function, reduced pro-inflammatory cytokines, increased BDNF levels	[95,96]
	Intranasal delivery of BDNF, PACAP38	Enhances synaptic plasticity	mouse models	Improved behavior, attenuated memory deficits, reduced HD neuropathology	[97,98,99]
Transcriptional dysregulation	Sodium butyrate	Modulates HDACs	R6/2 mice	Improved motor performance, extended survival, reduced HD pathology	[100,101]
	Synthetic molecules, such as 4b, RGFP966	Promotes pyruvate dehydrogenase activity, improves mitochondrial dysfunction	Transgenic mice	Improved motor performance, reduced striatal atrophy	[102,103,104]
	Selistat, nicotinamide	HDAC inhibition, SIRT1 inhibitor	Transgenic mice	Improved motor performances, increased BDNF levels	[105,106,107]

Acronyms: AMPK—5’ AMP-activated protein kinase; SIRT1—sirtuin 1; PGC-1α—peroxisome proliferator-activated receptor gamma coactivator 1-alpha; Nrf2—nuclear factor erythroid 2–related factor 2; mTOR—mammalian target of rapamycin; EAAT2—excitatory amino acid transporter-2; NMDAR—N-methyl-D-aspartate receptor; PPAR—peroxisome proliferator-activated receptor; JNK—c-Jun N-terminal kinase; BDNF—brain-derived neurotrophic factor; MAPK—mitogen-activated protein kinases; ERK—extracellular signal-regulated kinase; PACAP38—pituitary adenylate cyclase-activating polypeptide 38; NF-κB—nuclear factor-kappa-light-chain-enhancer of activated B cells; HDAC—histone deacetylase.

**Table 3:** Chemical modifications of ASOs with resultant characteristics and mechanism of action.

Modification	Resulted ASO	Main Properties	Mechanism of Action	References
Backbone modifications	Phosphorothioate (PS)	− Increased protein binding and cellular uptake − Increased resistance to nucleases	Degradation of mRNA by RNase H	[138]
	Phosphoroamidate (PA)	− Enhanced nuclease resistance	Non-degrading RNA mechanisms	[139]
	Phosphorodiamidate morpholino oligomers (PMO)	− Improved nuclease and protease resistance	Non-degrading RNA mechanisms	[140]
	Peptide nucleic acids (PNA)	− Increased nuclease and protease resistance	Non-degrading RNA mechanisms	[141]
Sugar modifications	2′-*O*-methyl (2′-OMe)	− Enhanced nuclease resistance − Decreased toxicity	Non-degrading RNA mechanisms and RNase activity with gapmer design	[142]
	2′-*O*-methoxyl-ethyl (2′-MOE)	− Enhanced nuclease resistance − Decreased toxicity	Non-degrading RNA mechanisms and RNase activity with gapmer design	[143]
	Locked nucleic acids (LNA)	− Enhanced nuclease resistance − Decreased toxicity − Increased target affinity	Non-degrading RNA mechanisms and RNase activity with gapmer design	[144]
	S-constrained-ethyl (cEt)	− Decreased toxicity − Increased target affinity	Non-degrading RNA mechanisms and RNase activity with gapmer design	[145]

**Table 4:** Comparison of siRNA and shRNA therapeutic strategies.

	Small Interfering RNA (siRNA)	Short Hairpin RNA (shRNA)
Source	Exogenous source	Nuclear expression
Delivery methods	Via synthetic or natural polymers or lipids to the cytoplasm	Via viral or other vectors to the nucleus
Persistence	Short-lasted (rapid degradation)	Expressed for months to years
Required dosage	High	Low
Incidence of “off-target” effects	Higher than for shRNA approaches, higher immune activation, toxicity	Lower than for siRNA therapies, low toxicity and immune activation
Therapeutic applications	In acute diseases, where high doses can be tolerated without significant toxicity and which do not require lifelong frequent administrations	In chronic disorders, where low doses acting for a longer time are desirable

**Table 5:** Strengths and limitations of the novel therapeutic strategies in Huntington’s disease.

Target	Category	Molecule	Development	Strengths	Limitations
mHtt RNA	ASO	IONIS-HTTRx	Phase 3 clinical trial	Dose-dependent reduction of mHtt RNA and protein	Intrathecal delivery, requires multiple doses
		WVE-120102/120101	Phase 1b/2a trial	Allele-specific (rs362331/ 362307, do not lower wild-type Htt	Applicable only to the targeted SNP carrier, require multiple doses
		(CUG)7	Pre-clinical	Allele-specific, less reduction of wild-type Htt	Multiple dosing
	RNAi	AMT-130	Phase 1b/2a	Single dose	Allele-non-specific, requires intrastriatal viral vector-mediated delivery
		VY-HTT01	Pre-clinical	Single dose	Viral vector-mediated intracranial injection
	Small molecules	Branaplam	Pre-clinical	Oral administration	Multiple doses, lack of specificity, risk of off-target effects
		PTC518	Phase 1	Oral administration	Multiple doses, lack of specificity, risk of off-target effects
DNA	ZFP	TAK-686 ZF-KOX1	Pre-clinical	Prevents mHtt transcription without altering the gene itself, single dose	Viral vector, intrastriatal injection; can trigger immune reactions
	CRISPR/Cas9	Unnamed CRISPR/Cas9 molecules	Pre-clinical	Single dose; corrects genetic defect	Viral vector-mediated intracranial delivery; risk of off-target mutations
	TALEN	Still in development	Pre-clinical	Not known	Not known
Cell loss	Stem cells	Cellavita	Phase 2/3	Intravenous delivery	Inconsistent effects
		Fetal stem cell transplant	Phase 1	Single dose	Intrastriatal injection, risk of graft rejection
		Autologous stem cells	Clinical trial	Intravenous delivery	Uncertain cerebral penetration
Neuroinflammation	Monoclonal antibodies	ANX005	Phase 2	Intravenous delivery	Multiple doses required
		VX15/2503	Phase 2	Intravenous delivery	Multiple doses required
	Intrabodies	rAAV6-INT41	Pre-clinical	Prevents protein misfolding, promotes aggregate clearance	Intrastriatal injection, viral vector-mediated delivery

## Data Availability

Not applicable.
